# Correction: Biophysically grounded mean-field models of neural populations under electrical stimulation

**DOI:** 10.1371/journal.pcbi.1008717

**Published:** 2021-02-24

**Authors:** Caglar Cakan, Klaus Obermayer

In Fig 2, panels C and D are missing the points ‘B3’ and ‘B4’. Please see corrected Fig 2.

**Fig 2:**
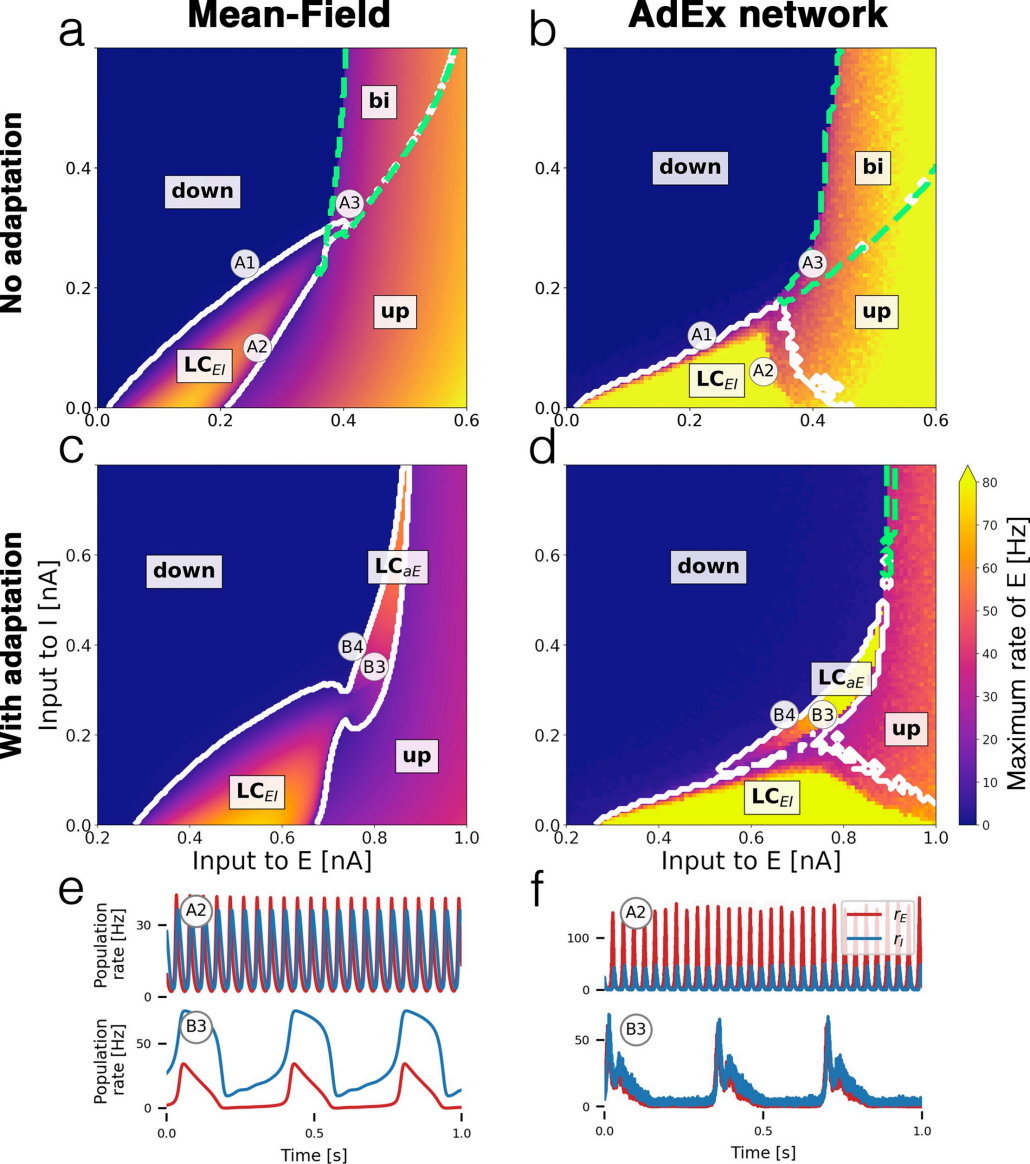
Bifurcation diagrams and time series. Bifurcation diagrams depict the state space of the E-I system in terms of the mean external input currents to both subpopulations *α* ∈ {*E*, *I*}. (a) Bifurcation diagram of mean-field model without adaptation with *up* and *down-states*, a bistable region *bi* (green dashed contour) and an oscillatory region LC_EI_ (white solid contour). (b) Diagram of the corresponding AdEx network with N = 50 × 10^3^ neurons. (c) Mean-field model with somatic adaptation. The bistable region is replaced by a slow oscillatory region LC_aE_. (d) Diagram of the corresponding AdEx network. The color in panels a—d indicate the maximum population rate of the excitatory population (clipped at 80 Hz). (e) Example time series of the population rates of excitatory (red) and inhibitory (blue) populations at point A2 (top row) which is located in the fast excitatory-inhibitory limit cycle LC_EI_, and at point B3 (bottom row) which is located in the slow limit cycle LC_aE_. (f) Time series at corresponding points for the AdEx network. All parameters are listed in Table 1. The mean input currents to the points of interest A1-A3 and B3-B4 are provided in Table 2.

Additionally, Eqs 11–14 provided in the Methods section contain minor transcription errors. Please see corrected Eqs 11–14.

$$\frac{d{\bar{s}}_{\alpha \beta }}{dt}=\tau_{s,\beta }^{-1}\;\left((1-\;{\bar{s}}_{\alpha \beta }(t))\cdot r_{\alpha \beta }(t)-{\bar{s}}_{\alpha \beta }(t)\right),$$ Eq (11)

$$\frac{d\sigma_{s,\alpha \beta }^{2}}{dt}=\tau_{s,\beta }^{-2}\;\left({\left(1-{\bar{s}}_{\alpha \beta }(t)\right)}^{2}\cdot \rho_{\alpha \beta }(t)\;+(\rho_{\alpha \beta }(t)-2\tau_{s,\beta }(r_{\alpha \beta }(t)+1))\;\cdot \sigma_{s,\alpha \beta }^{2}(t)\right),$$ Eq (12)

$$r_{\alpha \beta }(t)=\frac{c_{\alpha \beta }}{\left|J_{\alpha \beta }\right|}\;\tau_{s,\beta }\;K_{\beta }\cdot r_{\beta }(t-d_{\alpha }),$$ Eq (13)

$$\rho_{\alpha \beta }(t)=\frac{c_{\alpha \beta }}{\left|J_{\alpha \beta }\right|}\;\tau_{s,\beta }\cdot r_{\alpha \beta }(t).$$ Eq (14)
