# Supplementary figures and images for: Biophysically grounded mean-field models of neural populations under electrical stimulation
Source: PLoS Comput Biol. 2020 Apr 23;16(4):e1007822. doi: 10.1371/journal.pcbi.1007822 (PMC7200022; doi:10.1371/journal.pcbi.1007822)

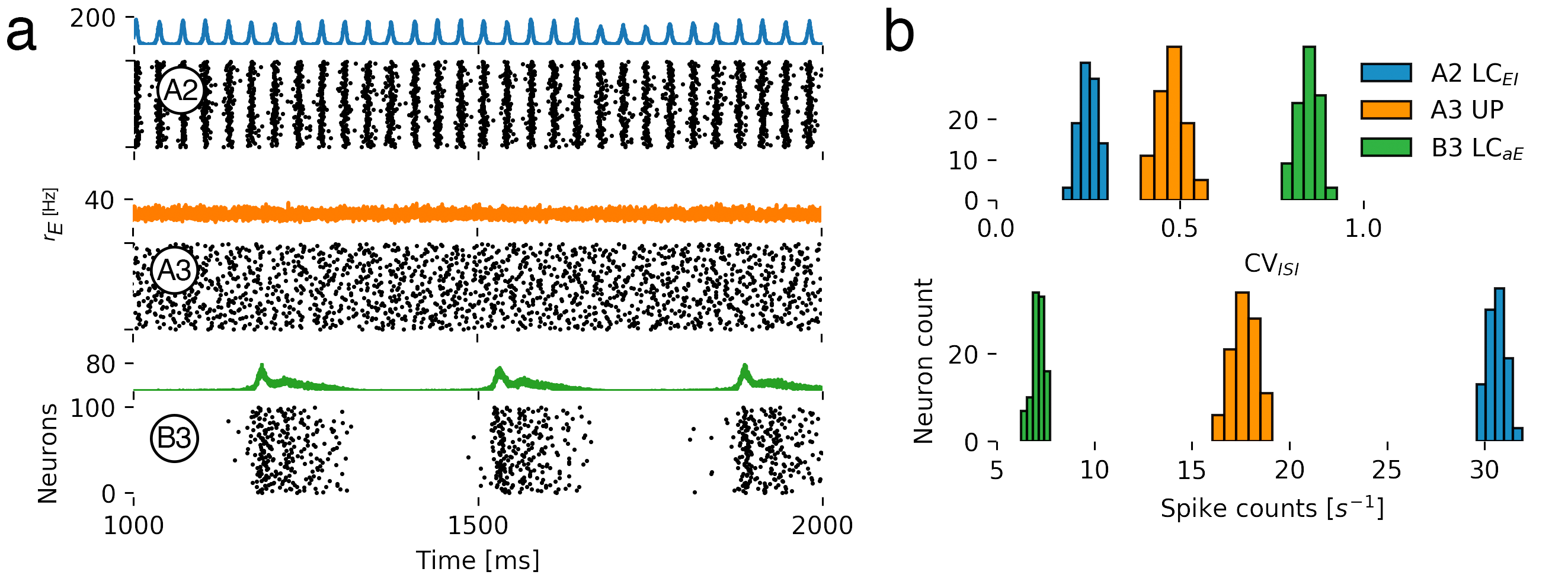

Supplement: S1 Fig — (a) Population firing rate rE of the excitatory population in Hz (upper panels) and raster plots of 100 randomly chosen excitatory neurons (lower panels) in three different network states A2, A3 and B3, located in the bifurcation diagrams Fig 2. A2 is located in the fast excitatory-inhibitory limit cycle LCEI, A3 in the high-activity asynchronous irregular up-state, and B3 in the adaptation-mediated slow limit cycle LCaE. (b) The upper panel shows the distribution of coefficients of variation (CV) of the inter-spike-intervals (ISI) calculated as the variance of ISIs divided by the mean ISI of excitatory neurons for all three states. The lower panel shows spike count distributions. For each neuron, the spike count was calculated from the inverse of the mean of the ISI distribution. Simulations were run with N = 100 × 103 neurons for 10s each. The statistics were computed for t > 500 ms for the neurons shown in (a). All parameters are given in Table 1. (TIF) [file pcbi.1007822.s001.tif]

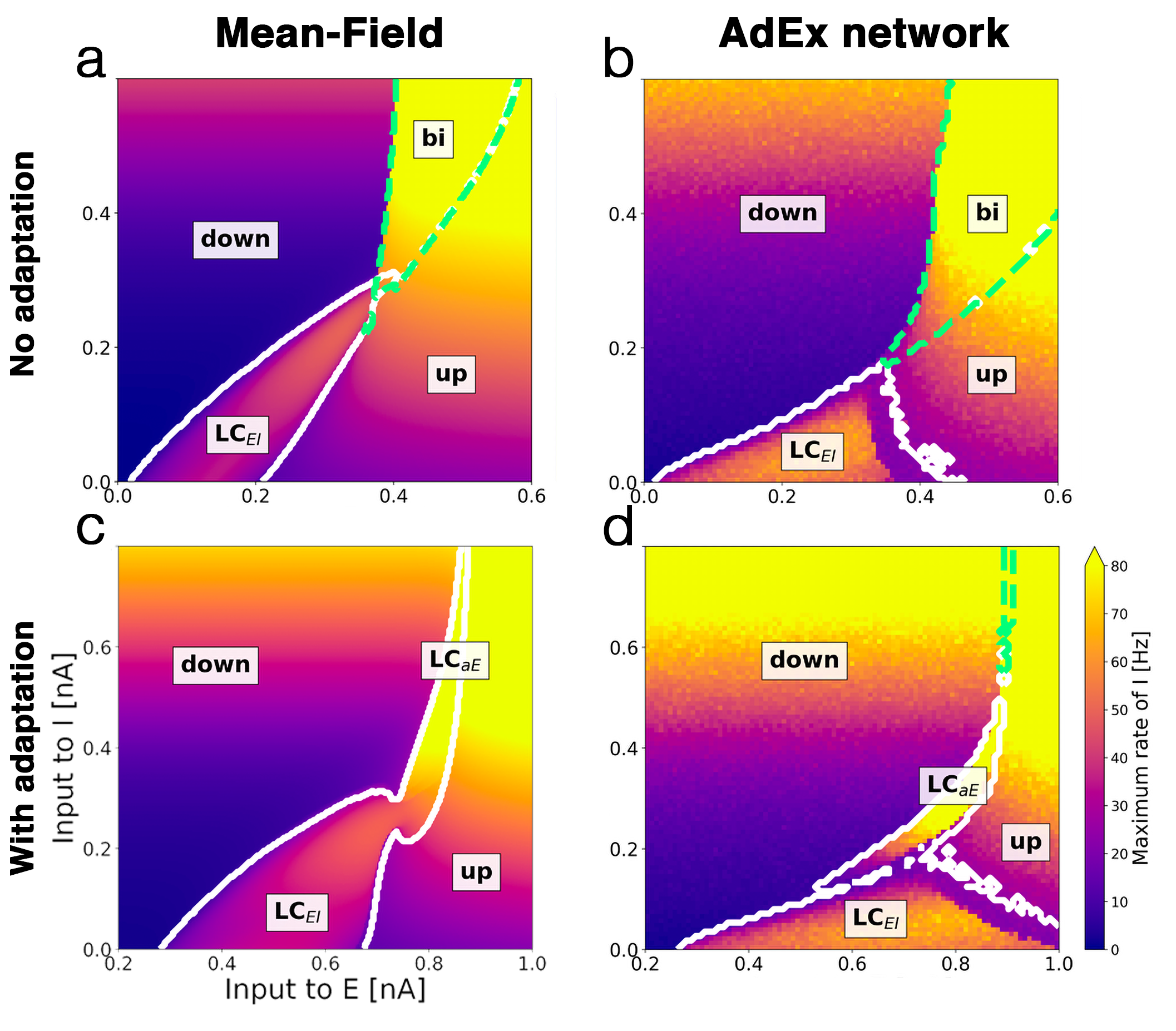

Supplement: S2 Fig — (a) Bifurcation diagram of the mean-field model without adaptation with up and down-states, a bistable region bi (green dashed contour) and an oscillatory region LCEI (white solid contour). (b) Diagram of the corresponding AdEx network. (c) The mean-field model with somatic adaptation has a slow oscillatory region LCaE. (d) Diagram of the corresponding AdEx network. The color indicates the maximum population rate of the inhibitory population (clipped at 80 Hz). All parameters are given in Table 1. (TIF) [file pcbi.1007822.s002.tif]

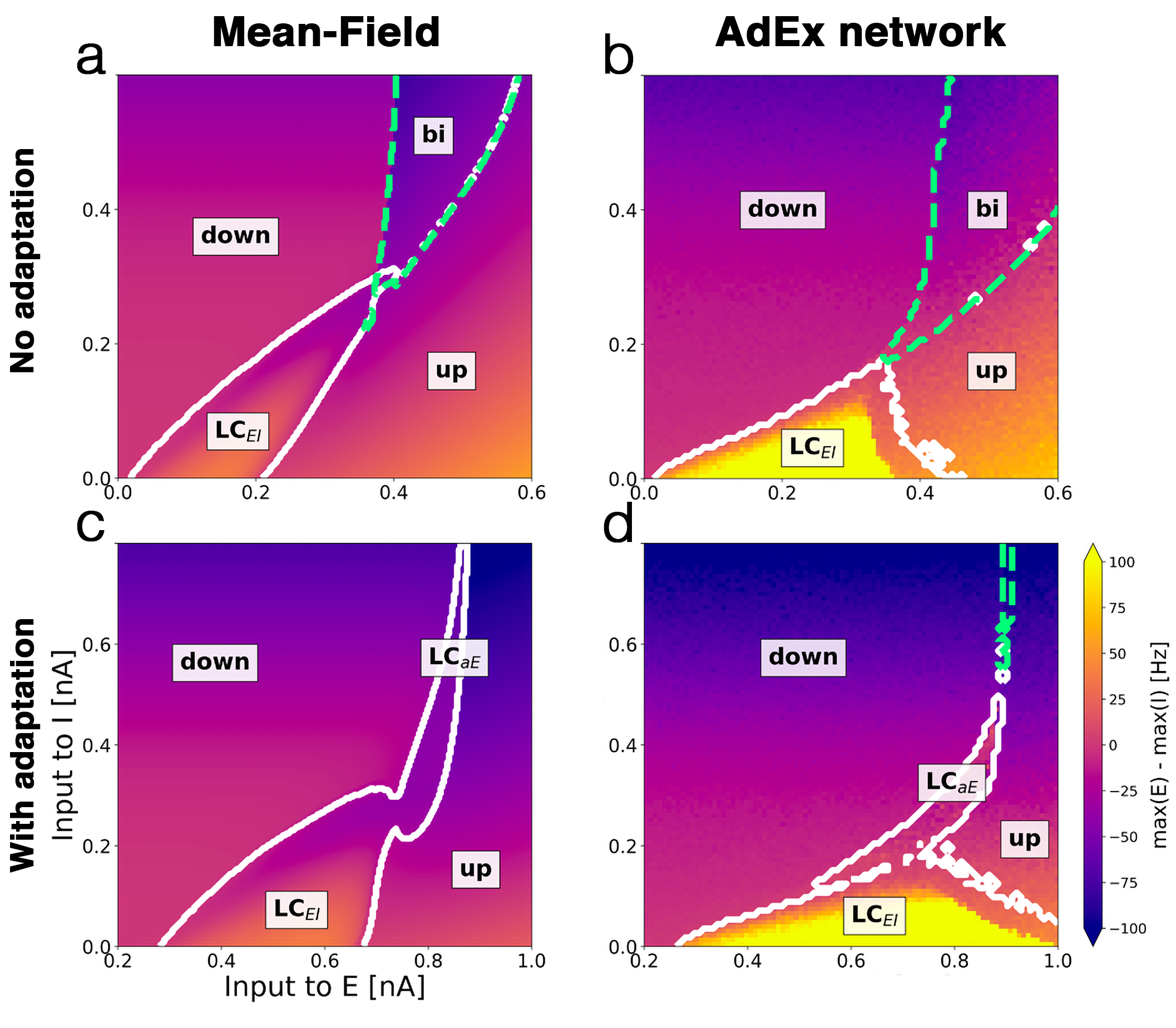

Supplement: S3 Fig — (a) Bifurcation diagram of the mean-field model without adaptation with up and down-states, a bistable region bi (green dashed contour) and an oscillatory region LCEI (white solid contour). (b) Diagram of the corresponding AdEx network. (c) The mean-field model with somatic adaptation has a slow oscillatory region LCaE. (d) Diagram of the corresponding AdEx network. The color indicates the difference of excitatory and inhibitory amplitudes (clipped from -100 Hz to 100 Hz). All parameters are given in Table 1. (TIF) [file pcbi.1007822.s003.tif]

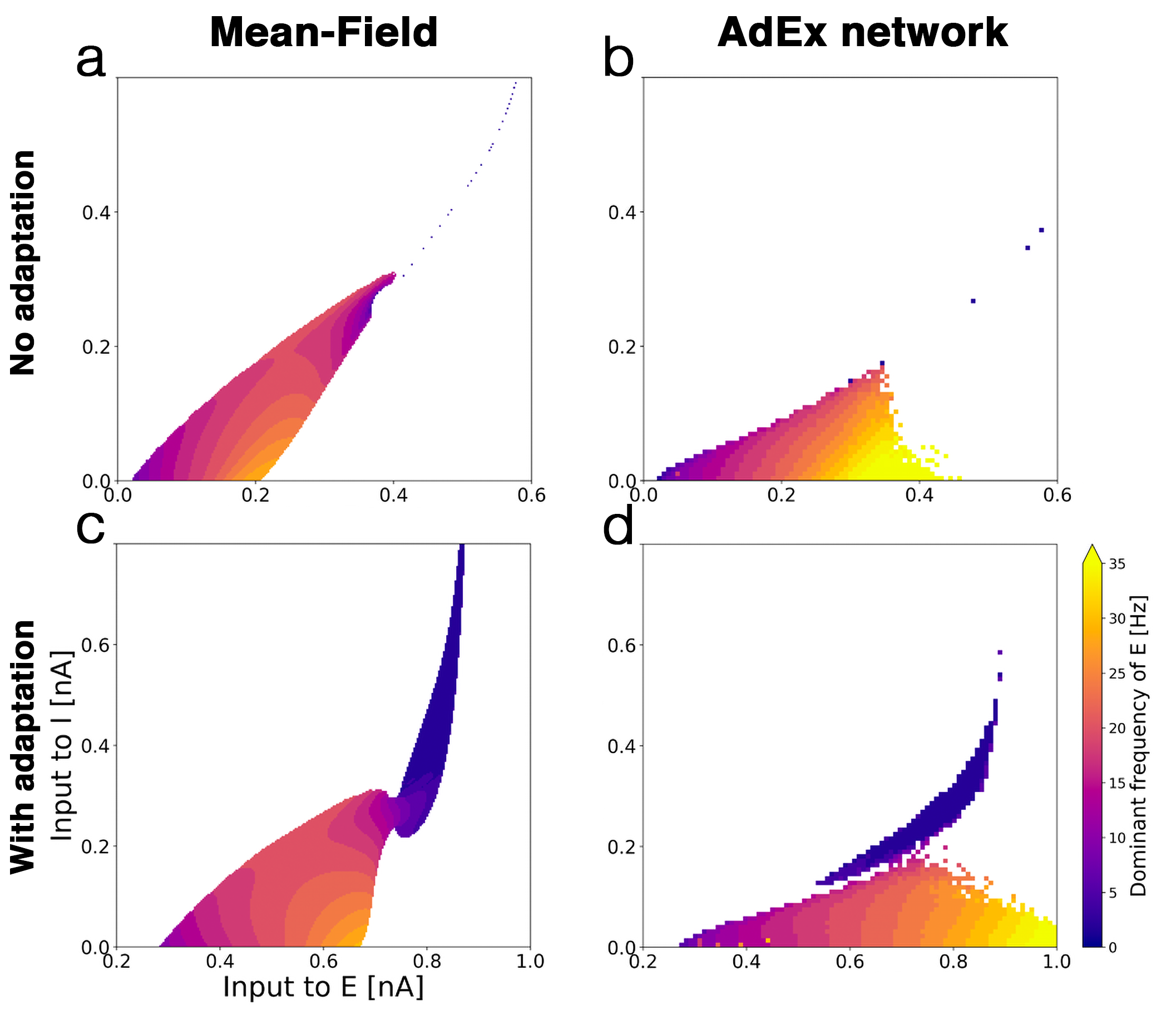

Supplement: S4 Fig — (a) Bifurcation diagram of the mean-field model without adaptation. (b) Diagram of the corresponding AdEx network. (c) Mean-field model with somatic adaptation. (d) Diagram of the corresponding AdEx network. The color indicates the difference of excitatory and inhibitory amplitudes (clipped at 35 Hz). All parameters are given in Table 1. (TIF) [file pcbi.1007822.s004.tif]

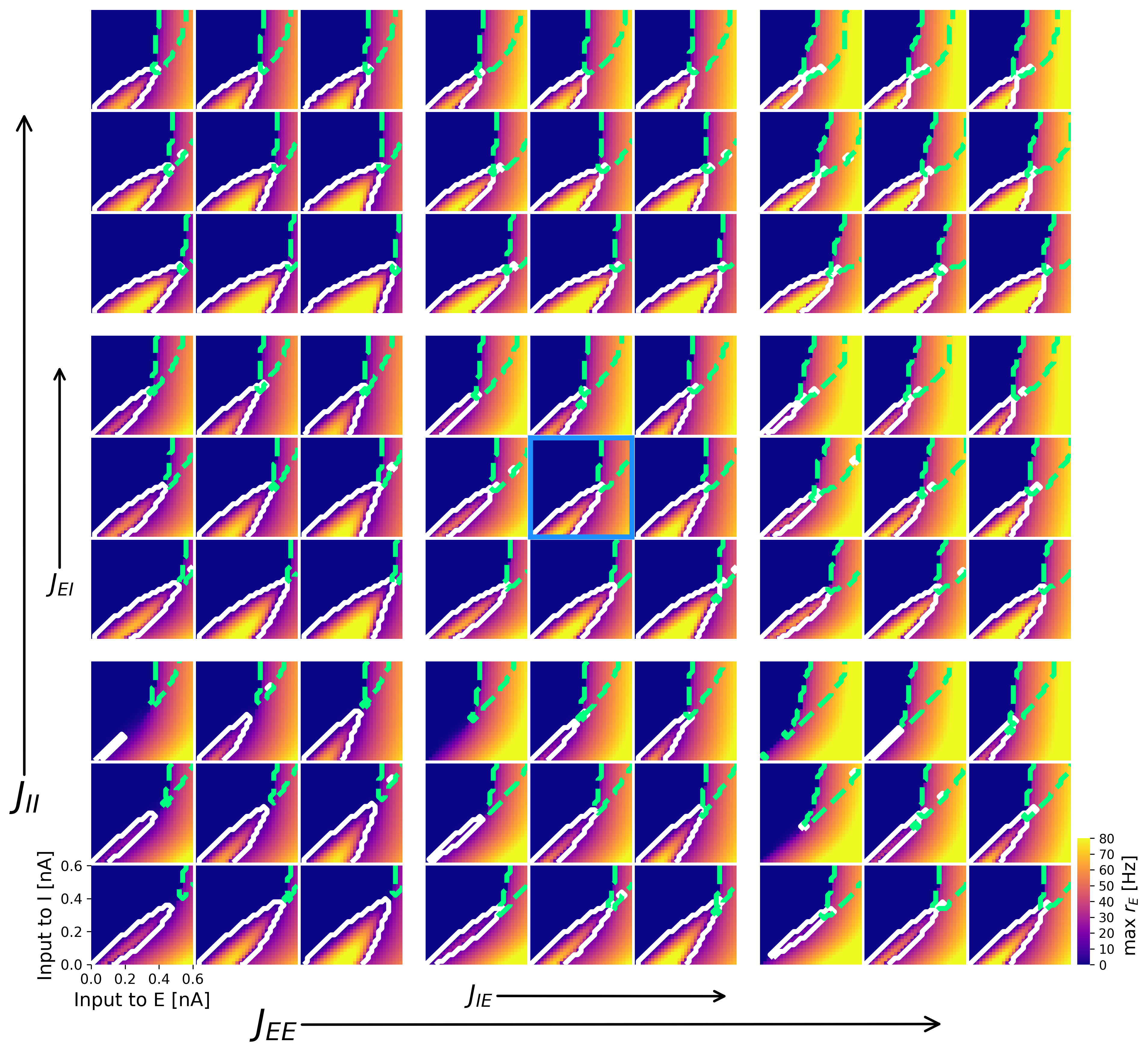

Supplement: S5 Fig — Stacked bifurcation diagrams depending on the mean input current to populations E and I showing dynamical states for intervals of JEE and JII (outer axis), JIE and JEI (inner axis) by values of 0.5 V/ms. The middle rows and columns correspond to the default value of the corresponding parameter (see Table 1). White contours are oscillatory areas LCEI, green dashed contours are bistable regions. Diagram in the middle (blue box) corresponds to bifurcation diagram Fig 2a. a = b = 0. For all other parameters, see Table 1. (TIF) [file pcbi.1007822.s005.tif]

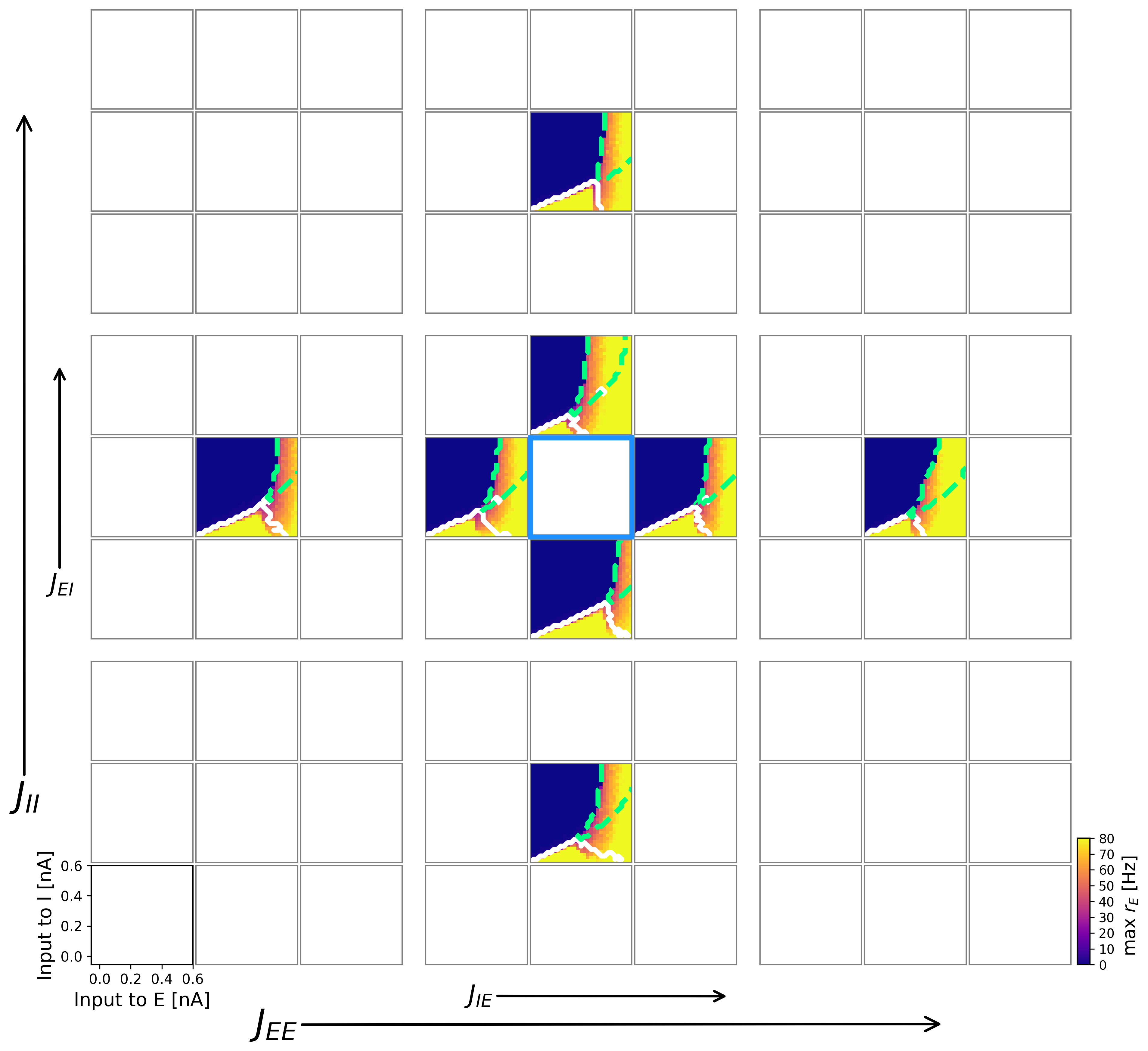

Supplement: S6 Fig — Stacked bifurcation diagrams for a subset of the values depicted in S4 Fig depending on the mean input current to populations E and I showing dynamical states for changing JEE and JII (outer axis), JIE and JEI (inner axis) by intervals of 0.5 mV/ms. The middle rows and columns correspond to the default value of the corresponding parameter (see Table 1). In this figure, all of the four coupling parameters have been varied independently. Empty plots were not computed. White contours within the plots denote the boundaries of the oscillatory areas LCEI, green dashed contours the boundaries of bistable regions. Position in the middle (blue box) corresponds to bifurcation diagram Fig 2b. Number of neurons N = 20 × 103, a = b = 0. For all other parameters, see Table 1. (TIF) [file pcbi.1007822.s006.tif]

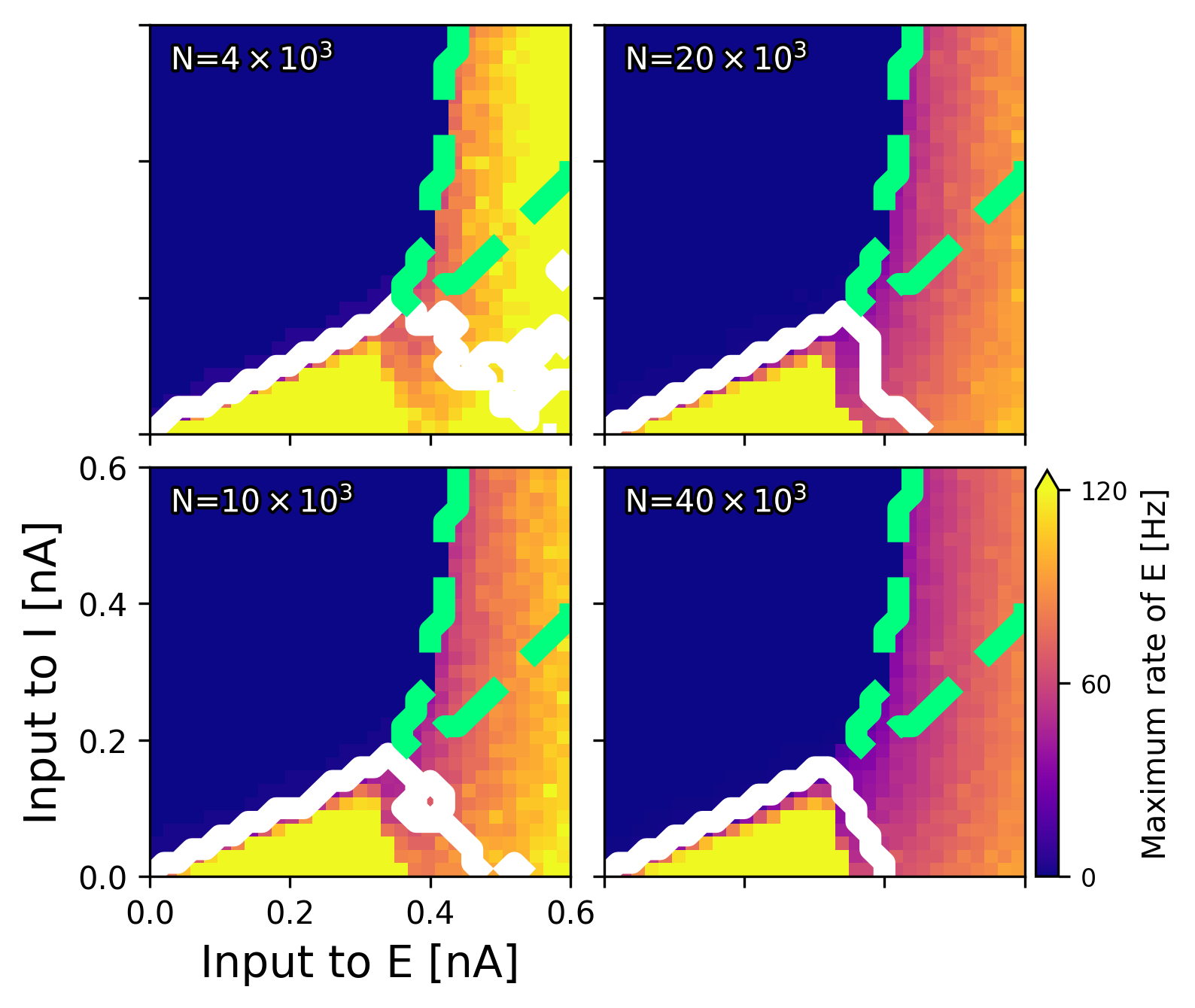

Supplement: S7 Fig — Bifurcation diagrams depict the state space of the E-I system without adaptation in terms of the mean external input currents C·μαext to both subpopulations α ∈ {E, I}. Up (bright area) and down-states (dark blue area), a bistable region bi (green dashed contour) and an oscillatory region LCEI (white solid contour) are visible. All parameters are given in Tables 1 and 2. (TIF) [file pcbi.1007822.s007.tif]

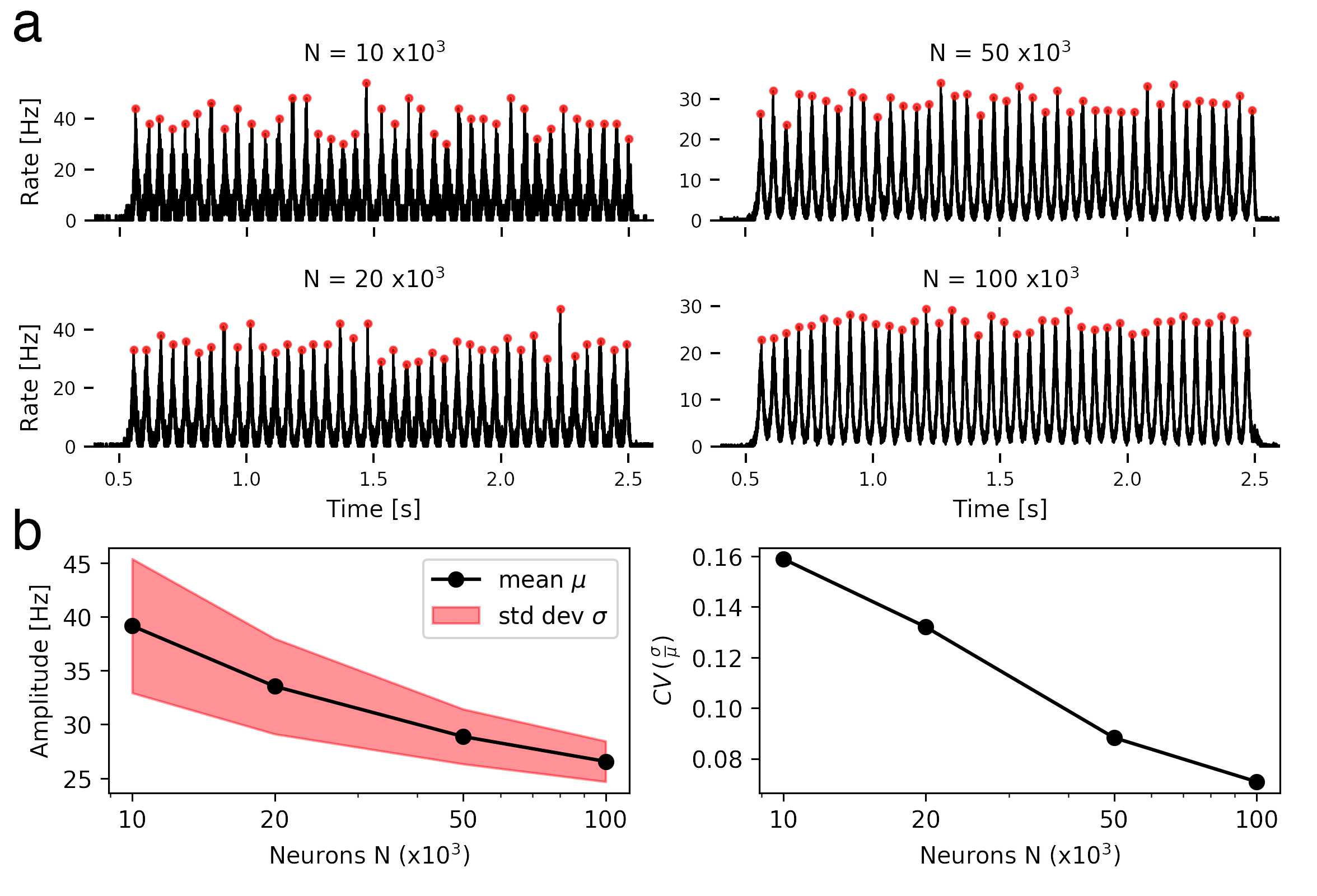

Supplement: S8 Fig — Oscillation amplitudes in the limit cycle LCEI fluctuate due to finite-size effects in the AdEx network. The system is parameterized in point A1 and pushed into the limit cycle by a constant input as in Fig 4b. (a) Traces of the population firing rates are shown (black) with the oscillation’s maxima marked (red dots) for an increasing number of neurons N in each panel (excitatory plus inhibitory). (b) The left panel shows the mean amplitude and the standard deviation as a function of the population size N on a semi-logarithmic scale. With increasing N, the amplitude of the oscillation decreases. The right panel shows the coefficient of variation (CV) of the amplitudes on a semi-logarithmic scale. The CV decreases with increasing number of neurons. Each point was measured from 20 realizations of 2 seconds of oscillatory activity. One randomly chosen realization for each N is shown in (a). All parameters are given in Tables 1 and 2. (TIF) [file pcbi.1007822.s008.tif]
