# Supplementary material for: Biophysically grounded mean-field models of neural populations under electrical stimulation
Source: PLoS Comput Biol. 2020 Apr 23;16(4):e1007822. doi: 10.1371/journal.pcbi.1007822 (PMC7200022; doi:10.1371/journal.pcbi.1007822)
Supplement: Attachment — Submitted filename: PLOS_response_to_reviewers.pdf [file pcbi.1007822.s009.pdf]

Dear Abigail Morrison and Lyle Graham, dear reviewers,

Thank you very much for the response to our submitted manuscript "Biophysically grounded mean-field models of neural populations under electrical stimulation" for review by PLOS Computational Biology. We also want to thank the reviewers for taking their time to carefully evaluate our work and making valuable comments on our manuscript. We have answered all the questions of the reviewers to our best abilities and have considered all comments to improve the quality of our manuscript. We hope to have addressed all issues raised appropriately.

Following your suggestions, we have integrated our research better into the existing experimental literature and added a self-contained paragraph on the limits of our theory to our Discussion section. We also present a stronger motivation of our work in the Introduction, better highlighting the significance of electrical stimulation in experiments. Moreover, our main theoretical and experimental questions are now clearly stated at end of our Introduction, additional to the Discussion section.

We found and corrected an error in the calculation of the equivalent electric field strengths in Figure 5 in the main manuscript, where we mistakenly used peak-to-peak amplitude values instead of zero-to-peak amplitudes, which we now use consistently. Using the translation function from electric field to electric current (Figure 8), we recalculated the electric field strengths that we report throughout our manuscript which fortunately leaves our conclusions unaffected.

Four new figures that are related to the questions of the reviewers were added to the Supplementary Material to extend our results and improve our manuscript. An extensive list of all changes can be found at the end of this document.

We are looking forward to hearing from you soon.

Best regards,

Caglar Cakan and Klaus Obermayer

Response to Reviewer #1

Issue 1

> *"The paper [...] assumes that the main effect of imposed electrical fields comes from the effect on dendrites, rather than axons [...]. See, for example, the work of Rattay [...]. This limitation should be discussed [...] and maybe it should be considered to change the title."*

As far as we are aware, the work of Rattay studies the effects of comparatively *strong and non-uniform* external fields which are generated by an electrode positioned close to the soma. In this case, the field effect scales with the second spatial derivative of the extracellular potential and the depolarization is strongest in the axon compared to other parts of the neuron. The stimuli in Rattay's work can cause up to 100 mV of membrane voltage change which can make the axonal segments generate action potentials [1, 2].

In our manuscript, we consider the case of a cortical mass in a *uniform and weak* electric field which is relevant for noninvasive stimulation techniques such as tACS *in vivo* or neuronal tissue immersed within two electrodes *in vitro* (such as in Ref [3]) that generates a uniform extracellular electric field. We now point this out more clearly in the Introduction. In this case, currents enter the cell at one end and leave it at the other, depending on the cell's alignment relative to the field [4]. Compared to Rattay's work, the external fields we consider are very weak and a DC field of 1 V/m increases the membrane potential of a single neuron by up to 0.5 mV from its resting potential in our model, which is in agreement with *in vitro* measurements [4]. Thus, the fields considered here are too weak to elicit a spike by themselves but can only modulate the spike timing properties of a single neuron, as it is shown in Ref. [5]. However, in the network, the effects on the population activity sum up considerably causing a significant influence of these weak electric fields, compatible with experimental observations [6].

Due to the uniformity of the field, the effect on a neuron largely depends on its angle of its main axis (defined by the direction of extension of the dendritic tree) to the electric field [4]. Although we expect that, in principle, axons could contribute to the somatic polarization due to a weak and uniform external field, most cortical axons are not geometrically aligned with each other the way that dendrites are organized in the columnar structure of the cortex [7] which would weaken the contribution of the axons to the overall somatic polarization of the population in a subthreshold field.

Despite the simplicity of the ball-and-stick model, it was shown in Ref. [5] that it can be used to reproduce the polarization behavior of a pyramidal cell in weak uniform fields. This was then translated into effective inputs to point neurons, such as the one used in our manuscript which lacks any morphological features.

To clearly communicate all of the assumptions and limits concerning our electric field model, we have now added a separate paragraph to our Discussion.

We believe the part of the title ”under electrical stimulation” is general enough, as it does not specify whether the stimulation effects considered in our manuscript come from electric current input or from an external electric field. We use the results from [5] to convert between the two quantities and report most of our results in both, input currents in units of pA and extracellular field strengths in units of V/m.

Issue 2

> *”I am also a bit uncertain about what is argued when it comes to electrical fields, are we only talking about externally imposed electrical fields or also ephaptic effects.”*

Throughout our manuscript, we speak of ”external fields”, which could could be generated by any arbitrary source, including endogenous fields of the brain. Our main focus, however, is put on fields generated during stimulation techniques such as tACS at experimentally and clinically relevant field strengths.

Indeed, the field strengths that we consider in our model are in the range of endogenous fields in the human brain, generated by the neural activity itself. Electrophysiological experiments show that ephaptic coupling can modulate subthreshold membrane voltages by up to 0.5 mV [8] and generate endogenous field strengths of more than 3.5 V/m [9]. Therefore, our findings actually support this observation from a theoretical perspective, since one of our main results is that considerable field effects are expected at these field strengths.

Currently, we do not have a straightforward method to incorporate the electric field *generated* by the population into our model yet. We certainly think that this is an interesting question and a possible extensions to our work in the future.

Issue 3

> *”As I understand it, the mean field (MF) model assumes very weak synaptic couplings ($c \ll 1$) [...]. However, in vivo synapses have been shown to have long tail distributions. This is a possible shortcoming of the model and the paper as it aims to be experimentally relevant.*

The Reviewer’s remark is valid in the sense that the approximation of using a small PSP amplitude c is a limitation of the theory, which is in a more general sense a mathematical property of the mean-field approach. In the *diffusion approximation*, we assume that an incoming spike to a neuron causes only small increases in the mean synaptic current, in order to replace the individual spike input to a neuron by a continuous variable representing the input rate and a Gaussian white noise term (as laid out in Ref. [10]). With increasing coupling strengths, this approximation may break down. To be more precise, we assumed

$c/J \ll 1$, which is a stronger assumption and which we state in our Methods now. Our values for c are chosen from previously used values in the mean-field literature [11] such that single spikes cause PSP amplitudes of 0.3 mV - 0.5 mV which agrees with *in vitro* measurements of pyramidal cells [12, 4]. Our approximation also preserves the assumption of $c \ll V_T - V_r$ that is typically found in spiking mean-field models without synaptic integration, for example in Refs. [10, 13].

Synaptic strengths are known to be log-normally distributed [14]. Other computational papers have specifically studied the effect of strong synapses on the population activity in Refs. [15] and [16]. Therein, they conclude that the incorporation of strong synapses causes the emergence of a new asynchronous state in which the firing rates of individual neurons fluctuate strongly, qualitatively different from the up-state that we observe. As a sanity check, we have computed the statistics of the spiking activity in our *up-state* and can confirm that in our case, the system exhibits the classical asynchronous state described in Ref. [17]. In Ref. [16], the author shows that firing rate models similar to ours break down and cannot capture the large fluctuations present in this state. In Ref. [15], a different model is used to specifically study the effect of log-normally distributed synaptic weights on the emergence of this new asynchronous state. If the mean of the coupling strengths is kept fixed, an increasing variance of the distribution of synaptic weights leads to an emergence of this highly irregular state for even weaker input values. We therefore conclude that 1) our mean-field model is limited to describing only weak synaptic coupling and that 2) incorporation of strong synapses leads to a new asynchronous state with large fluctuations in the single neuron's firing rates. We have now added a paragraph concerning this limitation of our model to our Discussion section.

> *"Simulations of the Mean Field model with varying coupling strengths are given in Fig S4, and it is mentioned in the discussion that bifurcation diagrams seems fairly robust. A comparison with the AdEx model would strengthen these claims and possibly shed light on differences."*

Following the suggestion, we have now computed a subset of the diagrams for the AdEx model in Fig S4 and show them here and in the Supplementary Figures.

In Fig. R1 it is visible that also the state space of the AdEx network model is fairly robust against similar changes of the coupling strengths, strengthening the fact that our operating point (middle panel of Fig. R1) is not specifically chosen to exhibit the kind of dynamics that we describe or optimized to fit the state space of the mean-field model. It remains that the mean-field model still produces a good approximation of the state space in this extended regime. We now mention this result in our manuscript.

> *"It would be interesting to see when/if similarities between AdEx and mean field brake when e.g. increasing synaptic couplings or making them heterogeneous."*

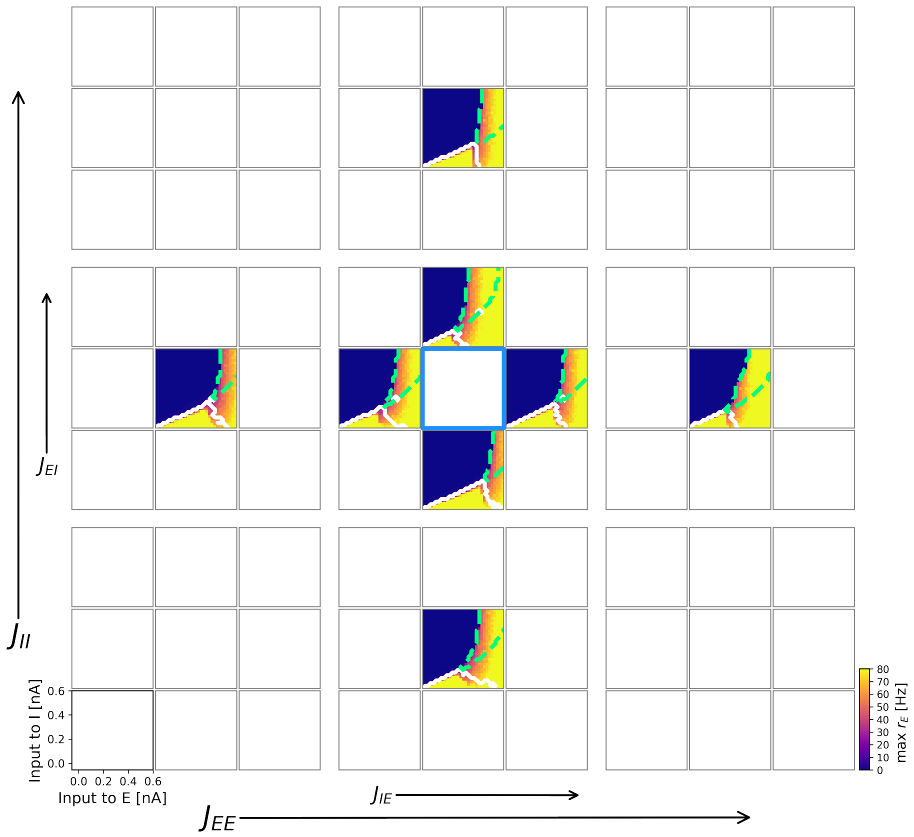

Figure R1: **Bifurcation diagrams of the AdEx network model for changing coupling strengths.** Stacked bifurcation diagrams for a subset of the values depicted in Fig. S4 depending on the mean input current to populations E and I showing dynamical states for changing J_{EE} and J_{II} (outer axis), J_{IE} and J_{EI} (inner axis) by intervals of 0.5 mV/ms. The middle rows and columns correspond to the default value of the corresponding parameter (see Table 1). In this figure, all of the four coupling parameters have been varied independently. Empty plots were not computed. White contours within the plots denote the boundaries of the oscillatory areas LC_{EI}, green dashed contours the boundaries of bistable regions. Position in the middle (blue box) corresponds to bifurcation diagram Fig. 2 b in the main manuscript. Number of neurons N = 20×10^3, $a = b = 0$. For all other parameters, see Table 1 in the main manuscript.

We agree that it is of value to analyse the differences of the mean-field model and the AdEx network more carefully. To stress this, we have now devoted a separate paragraph to the limitations of our model in our Discussion section. In the relevant ranges of parameters that we have explored (adaptation parameters, Fig. 3 in the main manuscript and synaptic coupling strengths, Fig. R1), we

could not find substantial mismatch of the mean-field model with the AdEx network, except the ones that we report in detail, such as the differences in the bifurcation diagrams Figs. 2, and in the time series Fig. 4. The authors in Ref. [18] have shown that heterogeneous synaptic strengths, drawn from a Gaussian distribution, can be incorporated in a straightforward way and the mean-field approximation is still in good agreement with the spiking network in this case.

However, the limits of the theory should become more apparent once the underlying assumptions are violated. One of these central assumptions is the large network assumption, namely for infinitely large networks, $N \to \infty$. To see what the effects of finite network sizes are, we have now computed new bifurcation diagrams of the AdEx network for smaller N (see Fig. R2). Even in these cases, the bifurcation diagrams largely coincide for as low as $N = 4 \times 10^3$ neurons, however the dynamics is dominated more and more by finite-size noise making it harder to clearly define the edges of the oscillatory region. We have added this additional result to our Supplementary Material and mention it in our manuscript.

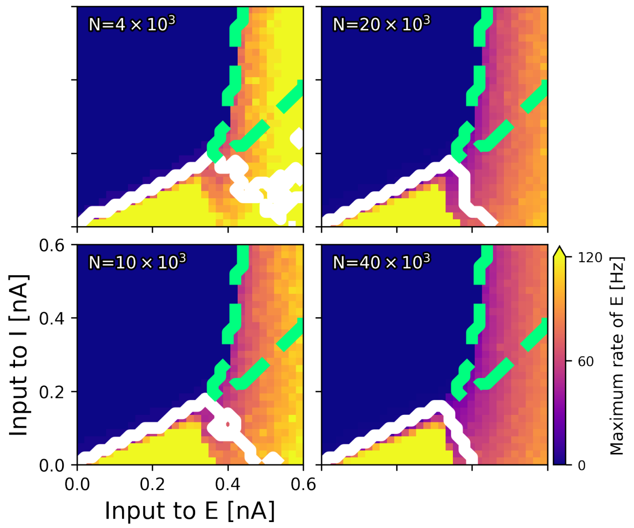

Figure R2: **Finite-size effects on bifurcation diagrams of the AdEx network with increasing number of neurons N.** Bifurcation diagrams depict the state space of the E-I system without adaptation in terms of the mean external input currents $C \cdot \mu_\alpha^{\text{ext}}$ to both subpopulations $\alpha \in \{E, I\}$. *Up* (bright area) and *down-states* (dark blue area), a bistable region *bi* (green dashed contour) and an oscillatory region LC_{EI} (white solid contour) are visible. All parameters are given in Table 1 and 2 in the main manuscript.

Issues 4

> *"In Fig 4. a-b there seems to be a beating in the AdEx firing rate not apparent in mean field model."*

The apparent modulation in Fig. 4 b is due to finite size noise. Although we used a very large number of neurons for this figure (N $= 100 \times 10^3$), the firing rate trajectories are not as smooth as in the $N \rightarrow \infty$ mean-field limit. To show that no additional frequencies are present in the system, we have simulated the same condition for a longer time and with different network sizes N and show frequency spectra in Fig. R3. As expected from a noisy oscillation, the spectrum widens around the peaks and the baseline power is increased with decreasing N. Since the modulation is due to finite size effects, we have analysed in more detail and show in Fig. R6 how the coefficient of variation (CV) of the amplitudes of this oscillation decreases and the AdEx network more closely resembles the dynamics of the mean-field model as the network size N increases. We have added this to our Supplementary Material and mention it in our manuscript in our Results section.

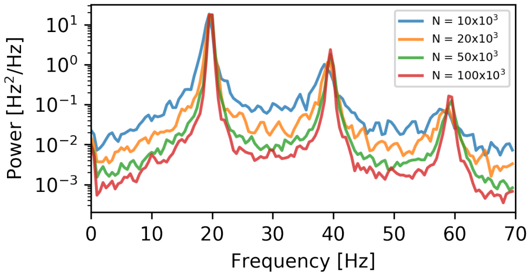

Figure R3: **Finite-size effects on frequency power spectrum of oscillations in the AdEx network.** The power spectrum of the oscillation in the fast limit cycle LC_{EI} is shown for different network sizes N. All parameters are as in Fig. 2b.

> *"In Fig 4. g-h there seems to be a re-bound after initial transient in the AdEx firing rate not apparent in mean field - this would also be interesting to visualize in frequency spectrum."*

The rebound visible in the AdEx firing rates in Fig. 4 h is a reflection of the fact that the up-state fixed point is actually a focus with a frequency associated to it and the same process is also visible in Fig. 4 f. This "ringing", or damped oscillation, into the fixed point from the initial overshoot is what is visible in the AdEx firing rates. The emergence of this oscillation can be explained by the fact that a common input transiently synchronizes many neurons that have been

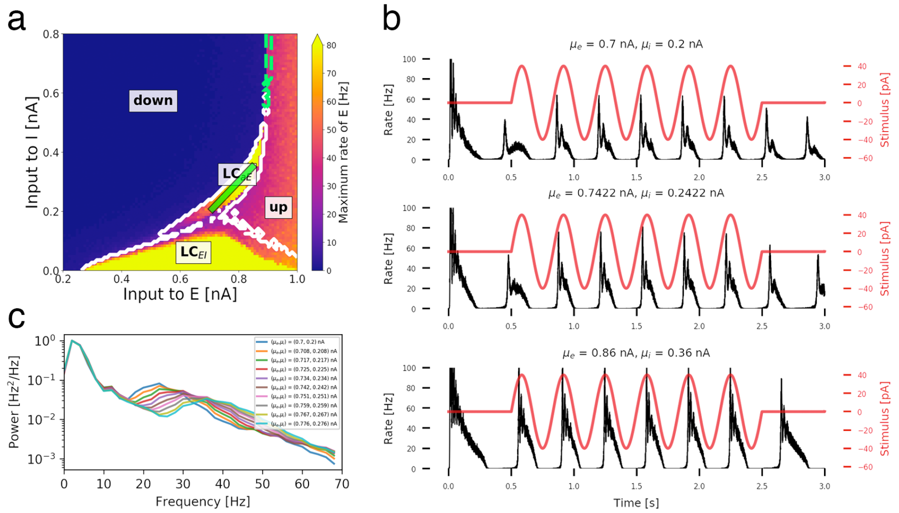

Figure R4: **Transient dynamics of down/up transitions in the AdEx network as in Fig. 4h.** **(a)** Bifurcation diagram of spiking neural network with adaptation as in Fig. 2 d in the main manuscript with a parameter trajectory (green bar) along the slow limit cycle **(b)** Frequency spectrograms along the trajectory show mainly a change of fast components of the oscillation **(c)** Time series of the population rate (black) and stimulus (red) as in Fig. 4 h for different parameters along the green line in (a).

asynchronous before. At the onset of the stimulus, the neurons fire together, leading to a sharp increase of the firing rate. Then, all neurons that have fired together, collectively undergo a refractory period, which causes the down-swing. This repeats until all neurons desynchronize again due to their independent noisy inputs and other sources of heterogeneity. This process can be seen in the rasterplot in Fig. R5 a, lower panel. We have analyzed the power spectrum of this oscillation and have chosen different parameters along the slow limit cycle in Fig. R4. We see that the frequency of the ringing activity changes along the limit cycle, while the dominant slow oscillation frequency stays constant, indicating successful frequency entrainment at the stimulation frequency.

This ringing at the onset of a steep input, was observed experimentally (see for example [19])) and has been a challenge to reproduce in mean-field models, such as in [20]. Our mean-field model only captures a very small part of this overshoot which is nearly invisible to see. One of such an overshoot can be seen in our manuscript in Fig. 4 c, where the transient from the oscillatory state to the *up-state* has a very small amplitude and is visible for only one oscillation period.

We believe that the reason our model only poorly reproduces these effects is due to the approximation of the linear response function of the AdEx network

by a decaying exponential (represented by a linear ODE in Eq. 7 with the timescale shown in Fig. 7c in the main manuscript), which itself doesn't produce any oscillations. A better approximation of this function could be a damped oscillation, as it was done in [13] to capture these transients and where it is shown that this oscillatory linear response function can capture over- and undershoots more closely. We now discuss this limitation in our manuscript and mention it as a possible extensions of our work.

> *"Fig 6 shows AdEx vs Mean field phase locking, but also with calculated external fields. However, it is mentioned earlier that fields may only be calculated without adaptation, are these results thus without adaptation i.e. the exponential integrate and fire model, not the AdEx?"*

This is correct. We observed similar phase locking behavior and Arnold tongues for the AdEx network with finite adaptation values. However, due to the limitations of the methods developed in Ref. [5], we can calculate the equivalent input currents only for EIF neurons (without adaptation), which is why we have included only the latter case in our manuscript.

Minor issues

> *"I couldn't find the point B2 (mentioned in the text) in Figure 2."*

B2 was a typo and was meant to be A2, which we now have fixed.

> *"What is meant by "the biophysical parameters of the AdEx network model are preserved in the mean-field description". I assume that there are not explicit formulas giving all mean-field parameters in terms of AdEx parameters?"*

Our goal was to highlight the fact that our mean-field approach (in contrast to so-called phenomenological models) does not introduce new and abstract parameters for the mean-field equations and the population description. Specifically, for a given set of single AdEx neuron parameters, we (pre-)compute transfer functions that are used in the mean-field model. All other parameters, such as input currents, network parameters and synaptic coupling strengths, as well as the parameters that govern the somatic adaptation mechanism and the membrane timescale and the synaptic timescale, are identical and directly represented in the equations of the mean-field model. This also allows for direct comparison of both models under changes of said parameters.

We have added a better explanation to our Methods Section and want to thank the reviewer for bringing this to our attention.

Response to Reviewer #2

Issue 1

> *"The work in this manuscript thus builds on the previous work in [13] and [5] but lacks a strong conclusion. This work could benefit from either a sound embedding in experimental literature or a more clear cut theoretical question"*

We have restructured our Discussion section with a better integration into experimental literature and a separate paragraph for each result that explains how our results relate to previous theoretical and experimental findings.

We also updated both Introduction and Discussion to highlight our main goals more clearly, which are twofold: our main theoretical goal is to assess the validity of the mean-field approach in a wide range of parameters and multiple populations with synapses, delays, and with non-stationary inputs in order to extend its validity to a more realistic case than it was done in Ref. [13]. Our results confirm that the mean-field model is in good agreement with results from network simulations.

The second, more experimentally relevant objective of our paper is to estimate the necessary field strengths to observe various electric field interaction effects. Our principal result here is that fields on the order of several V/m are able to affect the population rate considerably while the same field strengths have only a small effect on a single isolated neuron. This is well in line with experimental observations [4, 6] and indicates that field effects are strongly amplified in the network.

> *"The authors of [13] already showed that the mean-field model captures the oscillatory dynamics displayed by coupled populations of AdEx neurons."*

In Ref. [13], the authors considered only a single recurrent population at a time (excitatory neurons with adaptation *or* recurrent inhibitory neurons) whereas our cortical models consists of coupled excitatory and inhibitory neurons. Although isolated states with oscillations were studied in Ref. [13], the state space was not systematically explored. In Ref. [18], it was shown that adaptation causes oscillations in a mean-field model using the Fokker-Planck equation of recurrent E and I neurons, however the validity of these observations were checked on only a few points and the rest of the state space was not explored. In Ref. [13], the authors hypothesize about the relation between bifurcation parameters of the mean-field model and the AdEx network but parameters were not determined and validated.

One of our main goals was to study the interaction of oscillations and time-dependent external inputs. Therefore, we believe that our work is a solid extension of the work in Ref. [13] and our results are of interest for anyone studying the dynamics of a cortical E-I motif.

> *"This analysis also shows that stronger adaptation shrinks the region of bi-stability and replaces it with slow oscillations. It would be interesting to strengthen this nice mechanistic insight by theoretical analysis of the mean-field model. How does this result relate to other theoretical works of adaption?"*

The replacement of a bistability with a slow oscillation due to adaptation has been a focus of earlier theoretical works, including [21, 22, 23, 24, 25]. For example, in Ref. [24], the authors performed a linear stability analysis of the Fokker-Plank equation for two populations of LIF neurons. Their findings agree with our results: two stable fixed points are found in the inhibition-dominated region and the *up-state* loses its stability due to a Hopf bifurcation. Since our model is derived from a Fokker-Plank equation, we expect the mathematical properties of this transition to be very similar. However, the final mean-field model that we study is based on precomputed lookup tables (see Fig. 7 in the main manuscript), which does not allow for a purely analytical treatment. Our main goal was to show that the above-mentioned bifurcations also take place in the analogous spiking neural network and how this transition is represented in the state space.

To better relate our result to previous research, we have now included references to other theoretical works (in addition to the ones mentioned above) that focus on the interaction of adaptation with state switching between the resulting *up-* and *down-state* [21, 22] and how adaptation affects the intrinsic timescales of the network [26, 18] to our Discussion section.

> *"The authors state that realistic electrical currents can initiate transitions between dynamical states in the mean-field model. However, as far as I can tell, there is neither a discussion nor a reference to experiments and it is therefore not clear what these realistic current values are based on. Similarly, the statement that weak electrical inputs to the brain affect brain dynamics is not explored or substantiated further in the manuscript"*

In our Introduction and in our Discussion section, we refer to several experiments and reviews on modulation of brain oscillations using noninvasive tACS stimulation *in vivo* [27, 28, 29, 30, 31, 32, 33, 34] with relatively weak fields (around 1 V/m) as well as *in vitro* stimulation of neural populations with electric fields [3, 35, 36] (with higher amplitudes up to 8 V/m). Furthermore, we cite works that argue that endogenous fields, generated by the brain's activity itself, are able to entrain oscillatory activity of neighboring populations [9, 8] (with field strengths between 0.5 - 4 V/m). These experiments report electric field strengths at which several observations were made. In our Discussion, we refer to these amplitudes for each observed phenomenon (state switching, frequency entrainment and phase locking) and argue that they are compatible with the results of our model.

In order to embed our findings more clearly into the experimental context and cite all relevant works more visibly, we have restructured a significant part of our Discussion section in which we recapitulate our results.

Issue 2

> *"The work could be motivated in more detail."*

The main theoretical motivation for our work is to assess the validity of our mean-field approach and to prove that reduced models are useful for modeling time-varying electrical inputs. Our second main goal is to predict necessary field strengths for observing various stimulation effects that are relevant in experimental research. We now point our goals out more carefully at the end of our Introduction and relate them more clearly to the relevant experimental research in our Discussion.

Issue 3

> *"What is the motivation for analyzing frequency entrainment?"*

Frequency entrainment is an actively researched phenomenon in neural stimulation experiments [3, 35] and computational modeling [37, 38] which show that the amount of frequency entrainment depends on the stimulation amplitude and that the necessary amplitudes for frequency entrainment are higher than for phase locking. Our results confirm both of these observations.

In a broader sense, frequency entrainment is a more general phenomenon than phase locking: in order for two signals to have a constant phase relationship, the frequencies (or its harmonics) must to be similar. Therefore, experiments that study synchronization of neural oscillations often chose frequency-matched stimulation. Our results show that the frequency mismatch can be compensated up to a certain degree (i.e. frequency range) by choosing a stronger stimulation amplitude in order to achieve synchronization with the input.

Issue 4

> *"Why do the authors consider phase locking between input and population rate? Why at this particular stimulus frequency range?"*

Phase locking, sometimes referred to as "phase coherence" or simply "entrainment" in the literature, has been of significant interest in *in vivo* stimulation experiments. It was shown repeatedly that brain oscillations can be phase-locked to external stimuli and that this can affect information processing in the brain in various experiments [39, 29]. Particularly, sensory information processing depends on phase coherence of oscillations between distant brain regions [40, 41].

The chosen frequency range is dictated by the endogenous frequency of the system: in a small range around this frequency, and in an amplitude-dependent manner, the stimulus is able to lock the ongoing oscillation's phase. The resulting diagram in Fig. 6 is often referred to as an Arnold tongue.

Issues 5 and 6

> *"The authors could explore differences between the microscopic dynamics of the AdEx neurons and the mean-field model. How heterogeneous are the microscopic dynamics?"*

In order to answer this question, we present raster plots and statistics of the spiking neuron activity in three different network states in Fig. R5 and have added them to our Supplementary Material. In the oscillatory state in $\mathrm{LC_{EI}}$, in which the network oscillates at around 30 Hz, The neurons have a very low CV and the neurons fire in a mainly regular and synchronous state. In the up-state, the CV is higher and the spike counts between neurons vary more, indicating an asynchronous irregular state. Finally, in the slow oscillatory state $\mathrm{LC_{aE}}$ due to adaptation, the neurons have a much higher CV due to successive periods of rapid firing and no firing within the slow global oscillation. In this state, oscillations caused by adaptation can be seen as cycles of rapid and synchronous firing onset and slow asynchronous decline of the spiking activity.

Issue 7

> *"Are there finite-size effects?"*

Initially, for the simulation experiments in our manuscript, we have chosen the number of neurons to be high enough to minimize the effects of its finite size, ranging from $\mathrm{N} = 20\times10^3$ - 100×10^3 neurons. However, to answer the reviewer's question, we have conducted additional simulations to assess the magnitude of finite-size effects.

First, we computed new bifurcation diagrams of the AdEx network for smaller N (see Fig. R2). Even in these cases, the bifurcation diagrams largely coincide for as low as $N = 4 \times 10^3$ neurons, however the dynamics is dominated more and more by finite-size noise making it harder to clearly define the edges of the oscillatory region.

Furthermore, we measured the effect of finite-size noise on the coefficient of variation of the oscillation amplitude within the fast E-I limit cycle, visible in Fig. R6. With increasing network size N, the variance of the peak amplitude drops and the amplitude itself approaches the amplitude of the mean-field more closely. We have added both of these new results to our Supplementary Material and mention it in our manuscript.

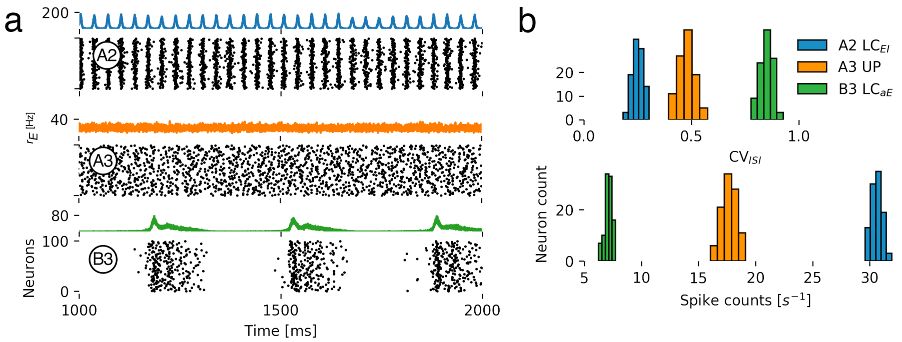

Figure R5: **Spiking network activity and statistics (a)** Population firing rate r_E of the excitatory population in Hz (upper panels) and raster plots of 100 randomly chosen excitatory neurons (lower panels) in three different network states A2, A3 and B3, located in the bifurcation diagrams Fig. 2 in the main manuscript. A2 is located in the fast excitatory-inhibitory limit cycle $\mathrm{LC_{EI}}$, A3 in the high-activity asynchronous irregular *up-state*, and B3 in the adaptation-mediated slow limit cycle $\mathrm{LC_{aE}}$. **(b)** The upper panel shows the distribution of coefficients of variation (CV) of the inter-spike-intervals (ISI) calculated as the variance of ISIs divided by the mean ISI of excitatory neurons for all three states. The lower panel shows spike count distributions. For each neuron, the spike count was calculated from the inverse of the mean of the ISI distribution. Simulations were run with $N = 100 \times 10^3$ neurons for 10 s each. The statistics were computed for $t > 500$ ms for the neurons shown in (a). All parameters are given in Table 1 in the main manuscript.

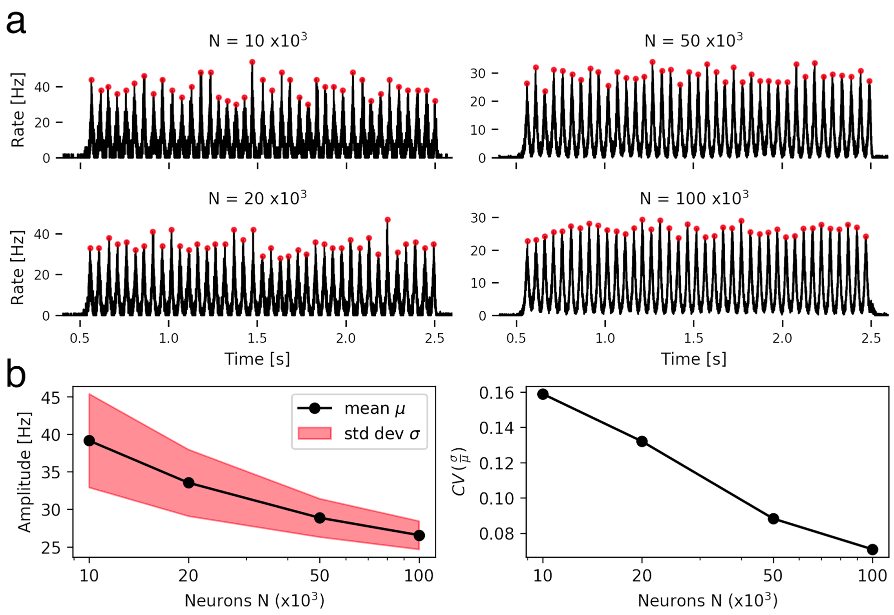

Figure R6: **Finite-size effects in the AdEx network on E-I oscillation amplitudes.** Oscillation amplitudes in the limit cycle LC_{EI} fluctuate due to finite-size effects in the AdEx network. The system is parameterized in point A1 and pushed into the limit cycle by a constant input as in Fig. 4 b in the main manuscript. **(a)** Traces of the population firing rates are shown (black) with the oscillation's maxima marked (red dots) for an increasing number of neurons N in each panel (excitatory plus inhibitory). **(b)** The left panel shows the mean amplitude and the standard deviation as a function of the population size N on a semi-logarithmic scale. With increasing N, the amplitude of the oscillation decreases. The right panel shows the coefficient of variation (CV) of the amplitudes on a semi-logarithmic scale. The CV decreases with increasing number of neurons. Each point was measured from 20 realizations of 2 seconds of oscillatory activity. One randomly chosen realization for each N is shown in (a). All parameters are given in Table 1 and 2 in the main manuscript.

Minor issues

> *"What is the number of neurons used in the simulation?"*

The number of neurons in the network simulations are listed in the Subsection "Numerical Simulations" in the Methods Section. Additionally, we now also mention the number of neurons in all of the relevant figure captions.

> *"The frequency entrainment for the mean-field and the AdEx network look quite different (Fig. 5c,d). It would be interesting to comment on that."*

The difference in the power spectra patterns is mostly due to the fact that the stimulus in the AdEx network was too weak to produce the same effects as for the mean-field model. This causes narrower entrainment frequency ranges and the system reverts back more easily to its endogenous frequency, producing horizontal lines in the diagram. We have now replaced the plot with a case with a slightly stronger stimulus, producing more similar entrainment patterns.

Even at these slightly stronger stimulus strengths, the remaining differences between the spectrogram of the AdEx network in Fig. 5 d and the mean-field model Fig. 5 c can be largely attributed to the fact that the AdEx network consistently needs stronger inputs to obtain the same effect as the mean-field model. This results in horizontal lines in areas where frequency entrainment is not effective and in faint and short diagonal lines between the lines that represent the (sub-)harmonics that are caused by interactions of the (sub-)harmonics. In the mean-field model, we mainly observe clear diagonal lines, indicating successful entrainment. Another source for the differences is the inherently noisy dynamics of the AdEx network, due to its finite size. We now comment on this more clearly in our manuscript.

> *"The time traces of Fig. 6c,d are zoomed out too far to see the phase locking."*

The dephasing of the input and the population activity happens at very slow timescales (over the course of seconds) which makes it hard to depict it in a readable plot. For this reason, we have plotted the Kuramoto order parameter below each time series to show that dephasing is actually happening. Following the reviewer's suggestion, we have now added zoomed-in insets to each time series that show a short time frame of the stimulus and the population activity, hopefully making the figure more accessible.

Moreover, we want to thank the reviewer for making us aware of typos that we have overlooked prior to submission which we have corrected in our updated manuscript.

List of all changes

Figures

- Added number of neurons N in all relevant figure captions (Figs. 2-6).
- Figure 5 d now showing frequency diagram for a stronger stimulus for better equivalence to Figure 5 c.
- Figure 5: All amplitude values are now measured from zero-to-peak.
- Figure 5: Equivalent field strengths are corrected accordingly.
- Figure 6 a and b: Equivalent field strengths are corrected accordingly.
- Figure 6 c and d: Time series now show a zoomed-in inset for better visibility.
- Figure 6: Updated caption.

Abstract

- Explicitly mention weak field effects in Abstract and Author summary.

Introduction

- Relation to *in vivo* tACS and *in vitro* stimulation in Introduction (Lines 6-8)
- Reference to enhancing memory consolidation with tACS (Line 11)
- Added alternative common abbreviation for AdEx: "aEIF" (Line 39)
- Clearly state the goals of the paper (Lines 56-61)
- Briefly summarize implications of our results (Lines 67 - 72)

Results

- Mention irregular firing activity of neurons and refer to new Supplementary Figure 1 (Line 110)
- Differences explained better between AdEx and mean-field model in Fig. 4 (Lines 188-194).
- More detailed explanation of the differences between Fig. 5 c and d (Lines 216 - 224)
- Corrected field amplitude values for Fig 6 (Line 250)

Discussion

- Major restructuring of the Discussion section.
- Added subsections for better readability.
- Add "same set of parameters for both models" statement (Lines 261-265)
- Clarify the bifurcation parameters and what they represent (Lines 273-275)
- Mention new result from Figure S6 (Lines 278-281)
- Reference new result Figure S1 (Line 282)
- Relation of our results to other theoretical works on adaptation (Lines 294-297)
- Report our results for DC fields and reference *in vitro* experiment (Lines 305-310)
- Briefly explain frequency entrainment (Lines 312-313)
- Report frequency entrainment results and reference *in vitro* experiments (Lines 316-320)
- Discuss relevance for ephaptic coupling (Lines 321-327)
- Reference *in vitro* observations of entrainment of (sub-)harmonics (Line 328-330)
- Experimental relevance of phase entrainment, embedding into literature (Lines 337-346)
- Summary of stimulation results and experimental relevance (Lines 347-355)
- Discuss weaker susceptibility of AdEx network (Lines 362-364)
- Discuss new results on finite-size effects (Lines 365-371)
- Frequency differences of AdEx and mean-field model (Line 372-373)
- Discuss limitation of our model: ringing activity at sharp transitions. Reference other mean-field models that focus on this (Lines 377-385)
- Discuss limitation of our model: weak synaptic coupling. Reference other theoretical papers that focus on this (Lines 386-397)
- Discuss limitation of our model: simplifying assumptions about field effects (Lines 398-410)
- Discuss possible axonal contributions (Lines 411-415)

- Discuss limitation of our model: assumption of weak fields (Lines 416-418)
- Discuss state-dependent effect of stimulation and relate to divergent experimental results in the literature (Lines 440-446)

Methods

- Assumption of weak synaptic coupling more specific (Line 454)
- Clarify "same set of parameters for both models" statement (Lines 505-512)
- Explain role of adaptive timescale in Eq. 7 (Lines 532-533)
- Removed "fast-spiking" (Line 608)
- Field effects only without adaptation (Line 688-690)
- Add reference for morphological neuron parameters (Line 704)
- Correct equivalent field strength effect (Lines 708, 712)
- Add reference for field effect size (Line 709)
- Explicitly mention identical number of neurons for both E and I (Line 721)
- Add reference to our code repository for easy reproduction of all results (Lines 731-735)

References

- New references were added.

Supplementary Figures

- Figure S1: New figure
- Figure S6: New figure
- Figure S7: New figure
- Figure S8: New figure

References

[1] Rattay F. Analysis of Models for Extracellular Fiber Stimulation. IEEE Transactions on Biomedical Engineering. 1989;36(7):676–682. doi:10.1109/10.32099.

[2] Rattay F. Analysis of the electrical excitation of CNS neurons. IEEE Transactions on Biomedical Engineering. 1998;45(6):766–772. doi:10.1109/10.678611.

[3] Reato D, Rahman A, Bikson M, Parra LC. Low-Intensity Electrical Stimulation Affects Network Dynamics by Modulating Population Rate and Spike Timing. Journal of Neuroscience. 2010;30(45):15067–15079. doi:10.1523/JNEUROSCI.2059-10.2010.

[4] Radman T, Ramos RL, Brumberg JC, Bikson M. Role of cortical cell type and morphology in subthreshold and suprathreshold uniform electric field stimulation in vitro. Brain Stimulation. 2009;2(4):215–228.e3. doi:10.1016/j.brs.2009.03.007.

[5] Aspart F, Ladenbauer J, Obermayer K. Extending Integrate-and-Fire Model Neurons to Account for the Effects of Weak Electric Fields and Input Filtering Mediated by the Dendrite. PLoS Computational Biology. 2016;12(11):1–29. doi:10.1371/journal.pcbi.1005206.

[6] Francis JT, Gluckman BJ, Schiff SJ. Sensitivity of Neurons to Weak Electric Fields. The Journal of Neuroscience. 2018;23(19). doi:10.1523/jneurosci.23-19-07255.2003.

[7] Mohan H, Verhoog MB, Doreswamy KK, Eyal G, Aardse R, Lodder BN, et al. Dendritic and axonal architecture of individual pyramidal neurons across layers of adult human neocortex. Cerebral Cortex. 2015;25(12):4839–4853. doi:10.1093/cercor/bhv188.

[8] Anastassiou CA, Perin R, Markram H, Koch C. Ephaptic coupling of cortical neurons. Nature Neuroscience. 2011;14(2):217–224. doi:10.1016/j.fsi.2017.07.004.

[9] Fröhlich F, McCormick DA. Endogenous Electric Fields May Guide Neocortical Network Activity. Neuron. 2010;67(1):129–143. doi:10.1016/j.neuron.2010.06.005.Endogenous.

[10] Renart A, Brunel N, Wang XJ. Mean field theory of irregularly spiking neuronal populations and working memory in recurrent cortical networks; 2004. Available from: `http://nba.uth.tmc.edu/homepage/cnjclub/2007summer/renart{\_}chapter.pdf`.

[11] Brunel N. What Determines the Frequency of Fast Network Oscillations With Irregular Neural Discharges? I. Synaptic Dynamics and

Excitation-Inhibition Balance. Journal of Neurophysiology. 2003;90(1):415–430. doi:10.4034/RBCS/2010.14.04.08.

[12] Williams SR, Stuart GJ. Dependence of EPSP efficacy on synapse location in neocortical pyramidal neurons. Science. 2002;295(5561):1907–1910. doi:10.1126/science.1067903.

[13] Augustin M, Ladenbauer J, Baumann F, Obermayer K. Low-dimensional spike rate models derived from networks of adaptive integrate-and-fire neurons: Comparison and implementation. PLoS Computational Biology. 2017;13(6). doi:10.1371/journal.pcbi.1005545.

[14] Buzsáki G, Mizuseki K. The log-dynamic brain: how skewed distributions affect network operations. Nature Reviews Neuroscience. 2014;15(4):264.

[15] Kriener B, Enger H, Tetzlaff T, Plesser HE, Gewaltig Mo. Dynamics of self-sustained asynchronous-irregular activity in random networks of spiking neurons with strong synapses. Frontiers in Computational Neuroscience. 2014;8(136):1–18. doi:10.3389/fncom.2014.00136.

[16] Ostojic S. Two types of asynchronous activity in networks of excitatory and inhibitory spiking neurons. Nature neuroscience. 2014;17(4):594–600. doi:10.1038/nn.3658.

[17] Brunel N. Dynamics of sparsely connected networks of excitatory and inhibitory spiking neurons. J Comput Neurosci. 2000;8(3):183–208. doi:10.1016/S0925-2312(00)00179-X.

[18] Augustin M, Ladenbauer J, Obermayer K. How adaptation shapes spike rate oscillations in recurrent neuronal networks. Front Comput Neurosci. 2013;7(9):1–11. doi:10.3389/fncom.2013.00009.

[19] Tchumatchenko T, Malyshev A, Wolf F, Volgushev M. Ultrafast population encoding by cortical neurons. Journal of Neuroscience. 2011;31(34):12171–12179. doi:10.1523/JNEUROSCI.2182-11.2011.

[20] Schwalger T, Deger M, Gerstner W. Towards a theory of cortical columns: From spiking neurons to interacting neural populations of finite size. PLoS Computational Biology. 2017;13(4):1–64. doi:10.1371/journal.pcbi.1005507.

[21] Moreno-Bote R, Rinzel J, Rubin N. Noise-induced alternations in an attractor network model of perceptual bistability. Journal of neurophysiology. 2007;98(3):1125–1139. doi:10.1152/jn.00116.2007.

[22] Destexhe A. Self-sustained asynchronous irregular states and up-down states in thalamic, cortical and thalamocortical networks of nonlinear integrate-and-fire neurons. J Comput Neurosci. 2009;27(3):493–506. doi:10.1007/s10827-009-0164-4.

[23] Jercog D, Roxin A, Barthó P, Luczak A, Compte A, De La Rocha J. UP-DOWN cortical dynamics reflect state transitions in a bistable network. eLife. 2017;6:1–33. doi:10.7554/eLife.22425.

[24] Tartaglia EM, Brunel N. Bistability and up/down state alternations in inhibition-dominated randomly connected networks of LIF neurons. Scientific Reports. 2017;7(1):1–14. doi:10.1038/s41598-017-12033-y.

[25] di Volo M, Romagnoni A, Capone C, Destexhe A. Biologically realistic mean-field models of conductance-based networks of spiking neurons with adaptation. Neural computation. 2019;31(4):653–680.

[26] Beiran M, Ostojic S. Contrasting the effects of adaptation and synaptic filtering on the timescales of dynamics in recurrent networks. PLoS Computational Biology. 2019;15(3):1–33. doi:10.1371/journal.pcbi.1006893.

[27] Reato D, Rahman A, Bikson M, Parra LC. Effects of weak transcranial alternating current stimulation on brain activity—a review of known mechanisms from animal studies. Frontiers in Human Neuroscience. 2013;7(October):1–8. doi:10.3389/fnhum.2013.00687.

[28] Thut G, Bergmann TO, Fröhlich F, Soekadar SR, Brittain JS, Valero-Cabré A, et al. Guiding transcranial brain stimulation by EEG/MEG to interact with ongoing brain activity and associated functions: A position paper. Clinical Neurophysiology. 2017;128(5):843–857. doi:10.1016/j.clinph.2017.01.003.

[29] Herrmann C, Rach S, Neuling T, Strüber D. Transcranial alternating current stimulation: a review of the underlying mechanisms and modulation of cognitive processes. Frontiers in Human Neuroscience. 2013;7:279. doi:10.3389/fnhum.2013.00279.

[30] Fröhlich F. Experiments and models of cortical oscillations as a target for noninvasive brain stimulation. Progress in Brain Research. 2015;222:41–73. doi:10.1016/bs.pbr.2015.07.025.

[31] Ozen S, Sirota A, Belluscio MA, Anastassiou CA, Stark E, Koch C, et al. Transcranial Electric Stimulation Entrains Cortical Neuronal Populations in Rats. Journal of Neuroscience. 2010;30(34):11476–11485. doi:10.1523/JNEUROSCI.5252-09.2010.

[32] Helfrich R, Rach S, Trautmann-Lengsfeld S, Schneider T, Herrmann C, Engel A. Entrainment of Brain Oscillations by Transcranial Alternating Current Stimulation. Current Biology. 2014;24(3):333–339. doi:10.1016/j.cub.2013.12.041.

[33] Witkowski M, Garcia-Cossio E, Chander BS, Braun C, Birbaumer N, Robinson SE, et al. Mapping entrained brain oscillations during transcranial alternating current stimulation (tACS). NeuroImage. 2016;140:89–98. doi:10.1016/j.neuroimage.2015.10.024.

[34] Huang Y, Liu AA, Lafon B, Friedman D, Dayan M, Wang X, et al. Measurements and models of electric fields in the in vivo human brain during transcranial electric stimulation. eLife. 2017;6:1–26. doi:10.7554/elife.18834.

[35] Deans JK, Powell AD, Jefferys JGR. Sensitivity of coherent oscillations in rat hippocampus to AC electric fields. Journal of Physiology. 2007;583(2):555–565. doi:10.1113/jphysiol.2007.137711.

[36] Vöröslakos M, Takeuchi Y, Brinyiczki K, Zombori T, Oliva A, Fernández-Ruiz A, et al. Direct effects of transcranial electric stimulation on brain circuits in rats and humans. Nat Commun. 2018;in Press. doi:10.1038/s41467-018-02928-3.

[37] Ali MM, Sellers KK, Frohlich F. Transcranial Alternating Current Stimulation Modulates Large-Scale Cortical Network Activity by Network Resonance. Journal of Neuroscience. 2013;33(27):11262–11275. doi:10.1523/JNEUROSCI.5867-12.2013.

[38] Herrmann CS, Murray MM, Ionta S, Hutt A, Lefebvre J. Shaping Intrinsic Neural Oscillations with Periodic Stimulation. The Journal of Neuroscience. 2016;36(19):5328–5337. doi:10.1523/JNEUROSCI.0236-16.2016.

[39] Neuling T, Rach S, Wagner S, Wolters CH, Herrmann CS. Good vibrations: oscillatory phase shapes perception. Neuroimage. 2012;63(2):771–778.

[40] Engel AK, Fries P, Singer W. Dynamic predictions: Oscillations and synchrony in top–down processing. Nature Reviews Neuroscience. 2001;2:704.

[41] Fries P. A mechanism for cognitive dynamics: neuronal communication through neuronal coherence. Trends in cognitive sciences. 2005;9(10):474–480.
