# Supplementary material for: Biophysically grounded mean-field models of neural populations under electrical stimulation
Source: PLoS Comput Biol. 2020 Apr 23;16(4):e1007822. doi: 10.1371/journal.pcbi.1007822 (PMC7200022; doi:10.1371/journal.pcbi.1007822)
Supplement: Attachment — Submitted filename: PLOS_review_2_answer.pdf [file pcbi.1007822.s010.pdf]

Dear Abigail Morrison and Lyle Graham,

Thank you very much for the response to our submission "Biophysically grounded mean-field models of neural populations under electrical stimulation". We are happy that we were able to address all issues appropriately so far.

We would like to address the minor issues that were raised by Reviewer #2:

> *"line 441: in order comprehend → to comprehend"*

We have corrected the typo.

> *"Figure R6: please add the mean-field prediction for the amplitude"*

Comparing the result of Figure R6 b (equivalently Figure S8 b in the Supplementary Material) to the prediction of the mean-field model is a good idea. However, a direct comparison of the amplitudes of the network simulation for a specific input parameter configuration to the predictions of the mean-field model with the same parameters is not straight-forward which is why we chose not to add them to this figure.

Throughout our manuscript, we have compared points in the state space of one model with *equivalent* points of the other model. As we mention, these points were not chosen to match the amplitudes or frequencies of both models but their location in the bifurcation diagrams. Simulations with even larger networks might yield that these differences vanish with increasing network size, however, we can't confirm this due to the vast amount of computing time this would require.

In Figure S8 b, we have plotted the oscillation amplitude of the network for one specific input parameter configuration at point A1. Since the bifurcation diagrams of both models are not perfectly equal, in the mean-field model, the point with exactly the same input parameters lies at a different location of the diagram when compared to the location of the limit cycle LC_{EI}.

A more complete comparison of the results from Figure S8 b to the mean-field amplitude predictions is already possible with the figures provided in our manuscript: In Figure 2 a, we have plotted the amplitudes of the mean-field model for all points in the oscillatory region.

Therefore, we would prefer to keep the Figure S8 b as is, because 1) the appropriate mean-field prediction for this point is not well-defined and 2) all information is already available in Figure 2.

We are looking forward to hearing from you soon.

Best regards,

Caglar Cakan and Klaus Obermayer

List of all changes

- "the other" $\rightarrow$ "another" (line 94)
- add "the system" (line 142)
- "that" $\rightarrow$ "which" (line 215)
- Fig6. caption: add "phase"
- add "producing Arnold tongues in the diagram" (line 240)
- "in order comprehend" $\rightarrow$ "in order to comprehend" (line 443)
- Methods: In Eq. 7, $\mu_\alpha^{\text{ext}} \rightarrow \mu_\alpha^{\text{ext}}(t)$
- Methods: "Here" $\rightarrow$ "In Eq. 7" (line 535)
- Remove redundant sentence "the mean-field model as well as the AdEx network, " (line 618)
- "indicates" $\rightarrow$ "means" (line 676)
- Fig 8. caption: add "equivalent"
- Edit reference to code for reproducing results (Methods/Numerical simulations, lines 731-735)
